# Effect of the nano/microscale structure of biomaterial scaffolds on bone regeneration

**DOI:** 10.1038/s41368-020-0073-y

**Published:** 2020-02-06

**Authors:** Lisha Zhu, Dan Luo, Yan Liu

**Affiliations:** 1 Laboratory of Biomimetic Nanomaterials, Department of Orthodontics, Peking University School and Hospital of Stomatology, National Engineering Laboratory for Digital and Material Technology of Stomatology, Beijing Key Laboratory of Digital Stomatology, Beijing, China; 2 State Key Laboratory of Heavy Oil Processing, College of New Energy and Materials, Beijing Key Laboratory of Biogas Upgrading Utilization, China University of Petroleum (Beijing), Beijing, China

**Keywords:** Regeneration, Mesenchymal stem cells, Bioinspired materials, Nanostructures, Regeneration

## Abstract

Natural bone is a mineralized biological material, which serves a supportive and protective framework for the body, stores minerals for metabolism, and produces blood cells nourishing the body. Normally, bone has an innate capacity to heal from damage. However, massive bone defects due to traumatic injury, tumor resection, or congenital diseases pose a great challenge to reconstructive surgery. Scaffold-based tissue engineering (TE) is a promising strategy for bone regenerative medicine, because biomaterial scaffolds show advanced mechanical properties and a good degradation profile, as well as the feasibility of controlled release of growth and differentiation factors or immobilizing them on the material surface. Additionally, the defined structure of biomaterial scaffolds, as a kind of mechanical cue, can influence cell behaviors, modulate local microenvironment and control key features at the molecular and cellular levels. Recently, nano/micro-assisted regenerative medicine becomes a promising application of TE for the reconstruction of bone defects. For this reason, it is necessary for us to have in-depth knowledge of the development of novel nano/micro-based biomaterial scaffolds. Thus, we herein review the hierarchical structure of bone, and the potential application of nano/micro technologies to guide the design of novel biomaterial structures for bone repair and regeneration.

## Introduction

**Fig. 1:**
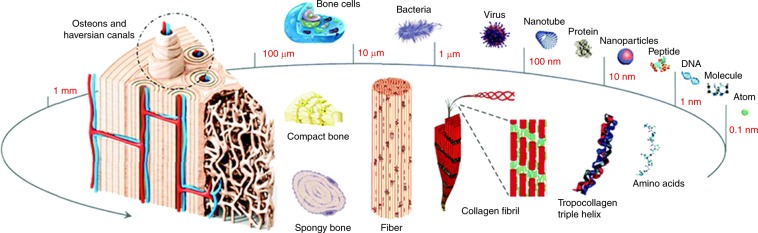
Hierarchical structure of bone tissue with various dimensions. (Adapted from ref. ^4^ with permission. Copyright 2017, Royal Society of Chemistry.)

Natural bone is a hard and dense type of connective tissue with excellent mechanical properties. It supports the human body, facilitates locomotion, protects internal organs, and stores and releases minerals. The excellent mechanics of native bone are closely correlated with its hierarchical structure from the nano to the macro scale, and precisely arranged inorganic and organic components at nanoscale: hydroxyapatite (HA) nanocrystals periodically deposit within collagenous gap regions during bone biomineralization (Fig. 1).^1–5^ The exterior structure of native bone (compact bone) consists of Haversian canals and osteons, while its internal part (spongy bone) has a trabecular structure of 75%–85% porosity.^6^ Natural bone contains four types of cells embedded in the extracellular matrix (ECM): osteoblasts, osteoclasts, osteocytes, and bone lining cells. Osteoblasts produce and mineralize new bone matrix, and repair older bone. Osteocytes are simply inactive osteoblasts trapped in the mineralized bone matrix, while osteoclasts are responsible for absorbing the matrix. Bone lining cells are inactive cells that are considered as precursors for osteoblasts. These cells have various roles in bone metabolism and ensure a balanced state in the context of dynamic bone remodeling.^7^ Furthermore, various cytokines, such as insulin-like growth factors, platelet-derived growth factors, fibroblast growth factors, vascular endothelial growth factors (VEGFs), transforming growth factors, and bone morphogenic proteins (BMPs) are sequestered in the bone matrix and regulate bone metabolism, function, and regeneration.^7,8^

Normally, bone has an innate capacity to heal from damage.^9^ However, self-repair is challenging when there are massive bone defects due to traumatic injury, tumor resection, or congenital diseases.^10^ Despite the emergence of scaffold-free tissue engineering (TE) as a powerful strategy using cell sheets, spheroids and tissue strands as building blocks, the use of biomaterial scaffolds remains the classical approach to regenerate bone due to the good degradation profile and advantageous mechanical properties, as well as to deliver important biomolecules (such as for the controlled release of growth and differentiation factors) or to immobilize them on the scaffold surface.^10,11^ Biomaterials mimicking the configuration of natural ECM can provide a bone-like microenvironment, facilitate stem cell recruitment, and regulate cellular behaviors in terms of cell adhesion, proliferation, migration and differentiation, and leverage the synergistic effect of cytokines for bone regeneration.^4^ Generally, the biofunctions of scaffolds depend on those of the biomaterial itself, as well as the complexity of processing conditions.^12^

Biomaterials can be categorized as bioactive ceramics, polymeric biomaterials, and composites. Bioactive ceramics can be of natural or synthetic origin, including coralline, bioactive glasses, calcium silicate, HA, tricalcium phosphate (TCP), and biphasic calcium phosphate (BCP). Bouler et al. reported the BCP scaffolds with multi-scale porosity and a composition of 87% HA and 13% β-TCP, exhibited a good bioactivity and positive effect on bone growth when implanted in porcine mandibular defects.^13^ As a matter of fact, ceramic biomaterials have some advantages such as good biocompatible as well as resistant to compression and corrosion, but their brittleness and low tensile strength need to be improved.^14^

The second category consists of natural polymers, such as chitosan (CTS), collagen (Col), fibrin, hyaluronic acid, and synthetic polymers including polycaprolactone (PCL), polyglycolic acid (PGA), polylactic acid (PLA), poly (vinylphosphonic acid) (PVPA). The structure and biochemical properties of natural polymers are much closer to those of the natural bone organic matrix. However, natural polymers have some unappealing performances, such as poor thermal stability.^15^ Similarly, synthetic polymers like PLA and PGA are not structurally ideal candidates as biomaterials for bone tissue regrowth due to the low osteoconductivity and compressive strength.^8^ In addition, an important class of polymers is represented by hydrogels, which are the most attractive ECM analogs. Specifically, natural hydrogels, whose polymers are based on natural sources, include Col, gelatin, agarose and alginate; synthetic hydrogels are fabricated using synthetic polymers such as polyvinyl alcohol, polyamides, and polyethylene glycol.^16^ These representative polymers can be usually fabricated by crosslinking macromers, polymerization of monomers or self-assembly of small molecules.^17^ Generally, increasing the mass concentration or crosslinking density of polymers is a logical way to improve their strength and stiffness. For instance, CTS/gellan gum ratio content into blends were reported to modulate the biofunctions of hydrogels such as cell adhesion, proliferation, and spreading. Furthermore, gelatin, produced through partial hydrolysis of Col, is mainly utilized for the production of microparticles, which are generally used as drug carriers. Raucci’s team developed two kinds of functionalized gelatin-based scaffolds through surface modification by HA nanoparticles and decoration with BMP-2, respectively.^18^ The scaffolds with inorganic contents improved cell attachment and early osteogenic differentiation in a short time, while the ones modified with the BMP-2 peptide tuned the biological response at long time.

Composites consist of a combination of two or more materials with different properties in the form of co-polymers, polymer–polymer blends, or polymer-ceramic composites, such as poly (lactic-co-glycolic acid) (PLGA), PCL/PVPA, PLA–HA, and CTS–calcium phosphate scaffolds, and soon.^19,20^ The composite biomaterials combine advantages of the above two scaffolds, and show good mechanical hardness and load-bearing capabilities as well as ideal biocompatibility. Lai et al. developed composite scaffolds of β-TCP and PLGA through 3D printing in order to increase mechanical stability and improve tissue interactions.^21^ The PLGA/TCP scaffold was reported to exhibit good biocompatibility, osteoconductivity, and biodegradability in vitro and in vivo studies. Besides, the addition of β-TCP or HA was demonstrated to improve the physical strength of hydrogels and enhance osteogenic differentiation and bone formation in vivo.^22^

Biomaterial fabrication methods include salt leaching, gas foaming, lyophilization technique, electrospinning, additive manufacturing (AM) technologies, and self-assembly.^7,22,23^ Thereinto, salt leaching, gas foaming and lyophilization techniques have been applied to produce porous scaffolds and change pore parameters while designing scaffolds. In the salt leaching method, pore sizes are controlled through using some porogens, such as wax, salt, and sugars; in the gas foaming technique, a porous structure is produced by using high-pressure carbon dioxide and controlling gas amount; while in the lyophilization technique, scaffolds are designed via the sublimation of the desired concentration of a solution.^22^ With advancements in technology and the onset of bio-inspired design principles, some innovative methods such as electrospinning, AM technologies and self-assembly have been widely applied to produce novel biomaterial scaffolds for bone TE. These fabricating methods are introduced in more detail later in the next section.

Recently, nano/micro-assisted strategies applied in regenerative medicine are becoming increasingly important. Nano/micro-materials including particles, composites and surfaces, provide a wide range of advanced approaches for bone regeneration.^24^ Based on the different spatial scales of biomaterial structures, they can be divided into nanoscale (≤100 nm), submicronscale (100 nm^–1^ μm) and micronscale (≥1 μm).^12^ Cao et al. demonstrated that the presence of 10% nanoparticles in a hydrogel enhanced the mechanical properties of the composite biomaterial and promoted new bone formation in animal.^25^ Zhang et al. magnetically labeled stem cells with Fe_3_O_4_ nanoparticles coated with nanoscale graphene oxide to form multilayered cell sheets in different patterns to induce bone formation.^26^ Liu et al. produced intrafibrillarly mineralized collagen with a bone-like hierarchical nanostructure (HIMC) under thermodynamic control. This biomimetic 3D collagen scaffold provided a good microenvironment for cell homing and multidifferentiation, and new bone formation.^27^ Its bone-like nanostructure, high porosity and interconnected pores favored the osteoblast cell migration and vascular ingrowth.

Herein, we review the architecture of natural bone, the various biomaterial fabrication methods, and the relationship between morphological and functional features to guide the design of biomaterial structures for bone repair. The spatial and temporal cues involved in the architecture of bone scaffolds at the nano/micro level are also discussed and we describe nano/micro-assisted strategies for bone regeneration.

## Fabrication of three-dimensional biomaterial scaffolds for bone regeneration

A highly interconnected porous architecture is the typical characteristic of native bone structure, which provides an ideal in vivo microenvironment to embrace abundant and diverse signaling cues influencing cell fate. Current bone repair biomaterial scaffolds are designed to reproduce such a microenvironment to promote cell ingrowth and differentiation, and vascularization for osteogenesis. Thus, biomaterial scaffolds having 3D hierarchical structures with porous nanostructures are the most promising bone substitutes.^28^ The defined structure, as a kind of mechanical cue, can influence cell behaviors and control some of their key features at the molecular and cellular levels.^7,12^ When fabricating bone substitute scaffolds, we need to consider the impact of physical cues such as internal porosity,^7^ pore structure, surface roughness, compressive moduli,^29^ and the alignment of ECM and bone cells.^30^

**Fig. 2:**
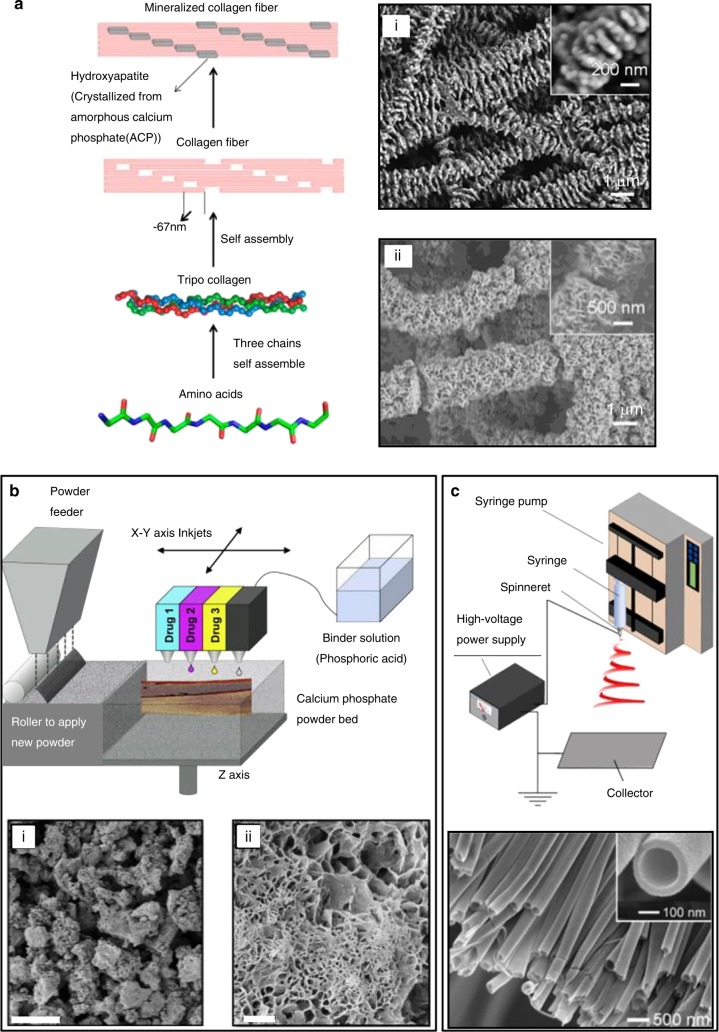
Typical schematic diagrams of self-assembly, 3D printing, and electrospinning. **a** Self-assembly process of mineralized collagen fiber formation (left panel) and scanning electron microscope (SEM) images of self-assembled, mineralized fibers (right panel) after 3 days (i) and 7 days (ii). **b** Low-temperature deposition manufacturing process (upper panel) and SEM images of internal pores of 3D scaffolds (lower panel). (i) Low magnification. Scale bar = 100 μm. (ii) High magnification. Scale bar = 10 μm; **c** Electrospinning process (upper panel) and a SEM image of electrospun nanofiber (lower panel). Inset: high magnification. (Reproduced with permission from refs. ^31–33^ Copyright 2013, American chemical society; Copyright 2014, Elsevier Ltd; Copyright 2017, American chemical society.)

Various methods have been applied to prepare 3D porous biomaterial scaffolds as bone substitutes. Specifically, the conventional techniques include solvent casting particle leaching (SCPL), thermally induced phase separation (TIPS), gas foaming, powder-forming, lyophilization and sol–gel science,^22^ while the new fabrication methods consist of self-assembly,^31^ AM^32^ and electrospinning (Fig. 2).^33^ In most circumstances, the realization of orderly hierarchical scaffolds depends on a combination of different methods, rather than on a single technology.^34^ Herein, we summarize common methods for fabricating scaffolds with hierarchical structures for bone repair, and review recent progress in this field.

### Solvent casting particle leaching

SCPL is one of the most widely researched techniques for preparing polymer-based porous 3D scaffolds for bone tissue regeneration due to the simplicity and approachable of this method without any requirement of expensive equipment.^35,36^ In principle, polymers are initially dissolved in an appropriate organic solvent to form a homogeneous polymer solution, and then the insoluble salt particles are admixed with the solution as pore-forming agents. The mixture can be further cast into a suitable mold, and a salt-polymer composite could be shaped after vacuum drying to remove the residual organic solvent. Porous architectures can be obtained after multiple rinses to remove salt particles. The process of SCPL brings many advantages: (i) the porosity and interconnectivity of the scaffold can be easily adjusted by regulating the proportion of salt particles and polymer; (ii) moreover, the pore size is also adjustable by selecting pore-forming agents with different geometric sizes. The selection of polymers needs to consider their biocompatibility, mechanical strength and biodegradability. To meet the above requirements, the polymers used are usually PLA, PGA and related copolymers.^37^ PCL, a semicrystalline polyester, has emerged as an idea candidate to fabricate long-term implants in bone TE due to its low degradation rate, excellent biocompatibility and relative high mechanical strength. For instance, Thadavirul et al. fabricated a highly interconnected, porous PCL scaffold by using the SCPL method, in which the sodium chloride and polyethylene glycol were used as porogens.^38^ The obtained salt- polyethylene glycol leached PCL scaffold possessed a uniform pore size of 378–435 μm, which leads to high water absorption capacity, and is conducive to culture mouse calvaria-derived preosteoblastic cells with high mineral deposition values. Wu et al. developed a zein/PCL biocomposite, in which the addition of zein improved the hydrophilicity, and also tailored the degradation rate of the scaffold.^39^ Although there are some concerns about SCPL methods, such as limited mechanical properties and inadequate pore interconnectivity, it could be inferred that the SCPL technique will become more sophisticated under the impetus of polymer science.

### Thermally induced phase separation

TIPS is another strategy to produce porous structures, which commonly involves following procedures. The biodegradable polymers should be initially dissolved in an appropriate solvent at elevated temperature. After removal of thermal energy, the phase separation occurs to form a polymer-rich phase and a polymer-lean phase. The porous polymer-based architecture could be obtained through freeze-drying of the phase-separated solution.^40–42^ The advantage of TIPS is that the density, dimension, morphology and interconnectivity of pores could be regulated by various parameters, such as polymer concentration, selection of solvent and additives, and quenching conditions. Blaker et al. used the TIPS method to fabricate highly porous PLGA microspheres with anisotropic channel-like morphology and ladder-like internal structure, which facilitates to drug delivery and further tissue regeneration.^43^ Lei et al. produced nanofibrous gelatin–silica hybrid scaffolds by using the TIPS technique.^44^ The acquired scaffold simulated bone ECM in terms of physical structure, chemical composition and biological functions. The gelatin–silica hybrid scaffold (with silica content of 30 wt%) possessed highest compressive modulus of (21.4 ± 8.2) MPa with porosity of 83.6% ± 0.8%. The in vitro experiments showed that the scaffold exhibited good biocompatibility and promoted cell proliferation.

### Sol–gel science

Sol–gel strategies, commonly used to synthesize bioactive glasses, have received much attentions in the field of TE due to the high surface area, good biodegradability and excellent osteoconductive properties of the sol–gel derived materials.^45–47^ The general process of sol–gel system consists of the following steps:^45^ firstly, the precursors (metal organic, inorganic compounds) are mixed with water to form sols after hydrolysis and condensation reactions; secondly, the sols are foamed, and start to condense after addition of surfactants and catalysts; thirdly, the foamed sols are transferred to a mold, and turn into gels after sealing for a while; last, the final thermal treatment densifies the matrix. Ding et al. synthesized polyhydroxybutyrate (PHB)/PCL/58S bioactive glass (60SiO_2_-36CaO-4P_2_O_5_, mol %) hybrid scaffolds by applying combined electrospinning and sol–gel techniques.^48^ The hybrid scaffold exhibits excellent physical and biological properties. The PHB/PCL blend matrix could improve biocompatibility and antibacterial ability with appropriate mechanical strength; meanwhile the bioactive glass greatly enhanced the hydrophilicity with the potential to upregulate osteogenic genes. The in vitro experiment indicated that the hybrid scaffold promoted the adhesion, viability and alkaline phosphate activity of MG-63 cells. Moreira et al. developed a CS–Col–bioactive glass nanoparticle hydrogel as a candidate of injectable TE materials.^49^ The addition of sol-gel derived bioactive glass nanoparticles could improve the scaffold stiffness significantly. Moreover, the hybrid hydrogel possessed a thermosensitive response under human temperature condition, and also exhibited good biocompatibility toward SAOS cell and HEK 293T cell.

### Gas foaming, powder-forming, and lyophilization techniques

Gas foaming, powder-forming, and lyophilization techniques are the simple and commonly used conventional methods to prepare 3D porous structures. The application of these techniques is relatively flexible, which can be used independently or combined with other methods. As literally, gas foaming is based on bubbling inert gas (such as nitrogen or carbon dioxide) into the precursor solution (polymer, ceramic, agar), transforming the liquid into foam. The generated foam is subsequently stabilized by lyophilization.^50,51^ The drawback of gas foaming is lack of precise control over the morphology of the scaffold. Costantini et al. developed a microfluidic foaming strategy to control insufflation of argon into a biopolymer aqueous solution. The acquired scaffold exhibited well ordered, crystal-like spatial arrangement of a porous structure.^52^ AliPoursamar et al. conducted a systematic research on how crosslinkers influence the porous gelatin scaffolds prepared via in situ gas foaming in terms of mechanical strength, microstructure, and cytotoxicity.^51^ The results showed that the longer crosslinking molecules improved the thermal stability of the scaffold with a more flexible structure and lower Young’s modulus. When referring to the powder-forming technique, it is always a broad concept. In general, chemically synthesized inorganic powders as bone substitutes are used to reshape to form 3D scaffolds after a series of treatments. The processing method could either be physical or chemical means, such as press forming and metallurgy processes. In recent years, the powder-forming has closely combined with AM (especially in 3D printing), which will be described in detail below. Lyophilization is a standard experimental method for removing solvents or volatile organic residues. It is also considered to be a versatile method to achieve the plasticity of a wide variety of materials.^53–56^ Although lyophilization has been proven to have little impact on the overall structure, the irreversible changes in the microstructure and ultrastructure prompt researchers to focus on the application of those lyophilized scaffolds for drug/factor delivery in TE.^57^

### Self-assembly

Self-assembly is the spontaneous process of molecules joining together to form a stable, structurally well-defined complex via noncovalent bonds, under equilibrium conditions.^58^ Self-assembly is ubiquitous in biological systems and regarded as the foundation for achieving complex biological structures.^58^ In TE, the self-assembly of biomaterials to mimic bone biomineralization (Fig. 2a) provides a bone-like microenvironment for proliferation and differentiation of osteoblasts, so that the substitute bone will have sufficient mechanical strength and stiffness.^1^ One of the most challenging aspects of the self-assembly of biomaterials is ensuring that the structural and biological functions of scaffolds are compatible with native bone.^59^ Some natural polymers, such as CTS, Col, silk and synthetic polymers including PGA, PLA, and PLGA are of particular interest due to their unique biocompatibility, biodegradability, immunogenicity, and versatility.^60^ Differing from polymers, bioactive glasses are often used for bone regeneration because of their mechanical properties and affinity to hard tissues. It is essential to develop a scaffold with enhanced osteogenic potential of the scaffolds.^60^ For instance, the porous biomaterial scaffolds developed by Quinlan et al. to encourage bone TE combined bioactive glass and collagen-glycosaminoglycan (CG) by using self-assembly and freeze-drying techniques.^61,62^ When bioactive glass particles were added to a CG slurry, a hydroxyl carbonate apatite layer was formed on the surface of the particles via ion exchange reactions of Na^+^ and Ca^2+^ with H^+^ or H_3_O^+^. The addition of bioactive glass could enhance the compressive modulus of the assembled composites, and VEGF production in endothelial cells.^61^ Col and HA also could be synthesized to form a Col-HA composites by dehydrothermal treatment and lyophilization in some studies.^27,63,64^ Furthermore, Liu et al. fabricated a high-performance bone-like hierarchical nanostructure with using a thermodynamic controlled self-assembly strategy involving two steps: fabrication of a high-energy polyacrylic acid-calcium intermediate and selective mineralization in collagenous gap regions driven by an energetically downhill process. The process was readily adjusted to different mineralization modes with distinct morphologies and biofunctions.^1,65,66^ Poly (acrylic acid) – calcium is important because it serves as a calcium transporter in the mineralization solution. This biomimetic bone-like scaffold with excellent biological performance had potent osteoinductive properties, which promoted mesenchymal stem cell (MSC) recruitment and induced macrophage polarization in osteogenesis, and furthermore assisted in new bone ingrowth by controlling the degradation rate.^27,65^

### AM technologies

AM, as a computer-aided fabrication technology, can make objects achieve rapid and seamless transition between a computer model and the physical realization thereof.^67^ During the process of design and fabrication, a computer-aided design 3D tissue model can be rendered mobile so that it can operate and guide working segments to move according to defined paths for the optimized carrier materials.^68^ Various methods and printing materials are used in conventional AM, such as stereolithography (SLA), fused deposition modeling (FDM), selective laser sintering (SLS), power-based 3D printing, 3D printing, extrusion deposition, and 3D bioprinting.^67^

The AM technologies are initially applied to fabricate composite parts with biologically-inspired architectures, such as brick-and-mortar patterns, reinforced periodic lattices, mechanically graded 3D geometries, well-defined surface topographies, rotating plywood designs and multilayered structures by using computer-aided design software.^69^ Correspondingly, these technologies can be used to produce 3D porous interconnected scaffolds and control the internal and external architecture readily.^22^ SLA is a rapid prototyping technique that uses photopolymerization to fabricate 3D scaffolds layer by layer according to a computer design program.^70^ Kim et al. tailored the scaffold design and optimized some structural parameters by SLA to obtain a proper porosity and pore size, in order to affect osteogenic cell signaling and ultimately in vivo bone tissue growth. FDM uses a movable head to create a physical object by depositing a thread of molten thermoplastic material onto a substrate. Porous polymeric bone scaffolds can be fabricated using the FDM technology with biocompatible and biodegradable materials to obtain appropriate elastic modulus.^71^ SLS is an AM technique that uses a high-power laser to melt thin layers of powder for structure production. In some studies, the metal or ceramic powder materials had been sintered directly from powder to bulk via SLS. Liu et al. fabricated a β-TCP/PLLA scaffold via SLS for bone repair, and introduced a transient liquid phase in SLS to improve compressive strength and fracture toughness of the TCP scaffolds.^72^ The dynamic powder-based 3D printing process involves selective solidification of various powders by different binders, which can be sprayed onto powder layers.^73^ Common materials used in powder-based 3D printing for bone regeneration include calcium phosphate (CaP) bioceramics, HA and TCP. Zocca et al. used powder-based 3D printing to shape mixtures of a preceramic polymer and fillers into complex porous scaffolds having a compressive strength of 1 MPa (for cylindrical scaffolds with a total porosity ~80%) and good bioactivity.^74^ However, the requirement for sintering of a conventionally printed powder-based 3D scaffold imposes severe constraints on the incorporation of bioactive molecules.^73^ Recent developments in LDM have enabled control of macropore size, interconnected micropores, and the incorporation of bioactive molecules (Fig. 2b).^75^ Unlike polymeric gluing at room temperature, 3D printing achieves material solidification via hydraulic setting reactions.^68,73^ A composite scaffold fabricated by LDM, with a designed microstructure and macrostructure had a high porosity (81.98% ± 3.75%), appropriately sized macropores and micropores size ((495 ± 54) μm and <10 μm, respectively) and good mechanical properties (compressive strength: (0.81 ± 0.04) MPa; elastic modulus: (23.14 ± 0.75) MPa).^76^ 3D printing has been employed to fabricate porous scaffolds by inkjet printing liquid binder droplets onto particulate matter. Because most biomaterials exist in either a solid or liquid state, a wide range of biomaterials has been utilized directly in the 3D printing. Lee et al. fabricate porous scaffolds with large pore sizes as well as scaffolds with fine features by the 3D printing technique to support cell growth in culture. This technique may be a useful adjunct for the fabrication of complex scaffolds for TE.^77^ Additionally, 3D printing via microextrusion can be performed using bioinks with modest-resolution materials, including hydrogels ceramics, Col, fibril, and silk.^78^ Bioinks must have suitable rheological properties and be capable of supporting cell growth and tissue development.^78^ An emulsion ink containing propylene fumarate dimethacrylate with a dense shell of PCL or PLA, was reported as bone graft; it had excellent biocompatibility, osteoconductivity, and enhanced mechanical properties (compressive modulus: ~15 MPa; yield strength: ~1 MPa).^79^ Last, 3D bioprinting is developed to fabricate 3D functional living human constructs suitable for clinical restoration of the functions of tissues and organs.^80^ It is the process of producing 3D multiphase tissue structures consisting of biomaterials, living cells and active biomolecules using AM technologies such us inkjet, extrusion, or laser AM technology.^81^ By printing stem cells into gel droplets and supplementing with growth factors, Gurkan et al. produced a multiphase tissue scaffold with osteogenesis and chondrongenesis potential, which could be used to repair bone–tendon tissue interfaces.^82^

AM is a promising bone reconstruction strategy that can readily produce 3D scaffolds having different shapes, internal structures, and mechanical properties. This strategy provides a rapid way to modify scaffold parameters and facilitate the fabrication of complex designs to elicit the desired physiological responses.^83^ Improved material properties and the incorporation of biological activities into novel scaffold designs could promote bone healing or anti-infection properties.

### Electrospinning

Electrospinning is a simple and accessible technique for the production of nanofibrils of various materials. The method can be used to improve the structure, porosity, surface, and alignment of nanofibers.^84^ Electrospinning is of interest because it can is process various polymeric materials to mimic the hierarchical architecture of the ECM, and manipulate cell behaviors for regenerative medicine (Fig. 2c),^33^ thereby favoring the infiltration and viability of cells.^43^ This solution-based approach relies on the electrostatic repulsion between surface charges to form nanofibers. In electrospinning, nanofibers are formed when a solution of a viscoelastic polymer is extruded through a stainless-steel needle at high voltage.^84,85^ This technique has been applied to prepare biomimetic and basic scaffolds from a variety of natural and synthetic biomaterials, such as Col, CTS, cellulose, HA, PLA, PC, and PCL/PLA blend solution.^84,86^ It has been shown that biomimetic electrospun HA/Col/CTS nanofibers could promote the osteogenic differentiation and bone regeneration in mouse cranial bone defect models. This biomimetic nanofiber system was prepared by first making a HA/CTS (30:70, w/w) nanocomposite via the coprecipitation, and then doping it with Col to provide multi-component solution for electrospinning.^87^ The function of nanofibers can be enhanced by manipulating their structure or adding other nanoparticles/bioactive molecules during the electrospinning process. Recently, bioinspired bone-like composite scaffolds, such as Col combined with the catecholamines and Ca^2+^,^85^ a VEGA-free polymeric scaffold,^88^ and BMP-2/poly(ε-caprolactone)-poly(ethylene glycol) co-polymer scaffold have been reported.^89^ Bioactive molecules loaded in a stable porous nanocarrier were dissolved in a copolymer solution containing polymeric nanofibers to yield the blending solution for electrospinning. These bioactive molecules mostly retained their bioactivities and achieved a sustained release to stimulate osteogenesis. Each electrospinning method has its own limitations in terms of biomaterial selection. Although the technique is an important route to fabricate bioinspired 3D scaffolds, further optimization is required for in vivo applications.

## Biological behaviors underlying nano/microstructure of biomaterial scaffolds

Bone tissue is a functionally and structurally graded system;^90^ long bone is a good example. In the macroscopic view, its cross-section can be divided into external compact cortical bone and internal spongy cancellous bone. The gradual structure change from cortical bone to cancellous bone and changes in the pore distribution determine a gradual change in mechanical properties, including tensile strength and elasticity.^90^ At the micro perspective, the organic and inorganic components mingle at the submicron scale to form mineralized collagen fibrils with staggered nanostructure.^91^ The hierarchical structures of bone range from the millimeter to the nanometer scale, e.g., fiber bundle (~1 μm), mineralized fibrils (~100 nm), and nanophases (collagen molecules and mineral particles).^1^ The close relationship, in terms of topography and construction, of the biomaterial with bone tissue accommodates the stress caused by the difference in stiffness between defect areas and biomaterials. Clearly, biomaterials require complex multiscale properties and abilities to influence cell behaviors at the molecular and cellular levels. Scaffold architecture is an important factor that guides or confines cell behaviors via direct contact. The following section reviews the importance of the scaffold nano/microstructure.

### From the nanoscale perspective

**Fig. 3:**
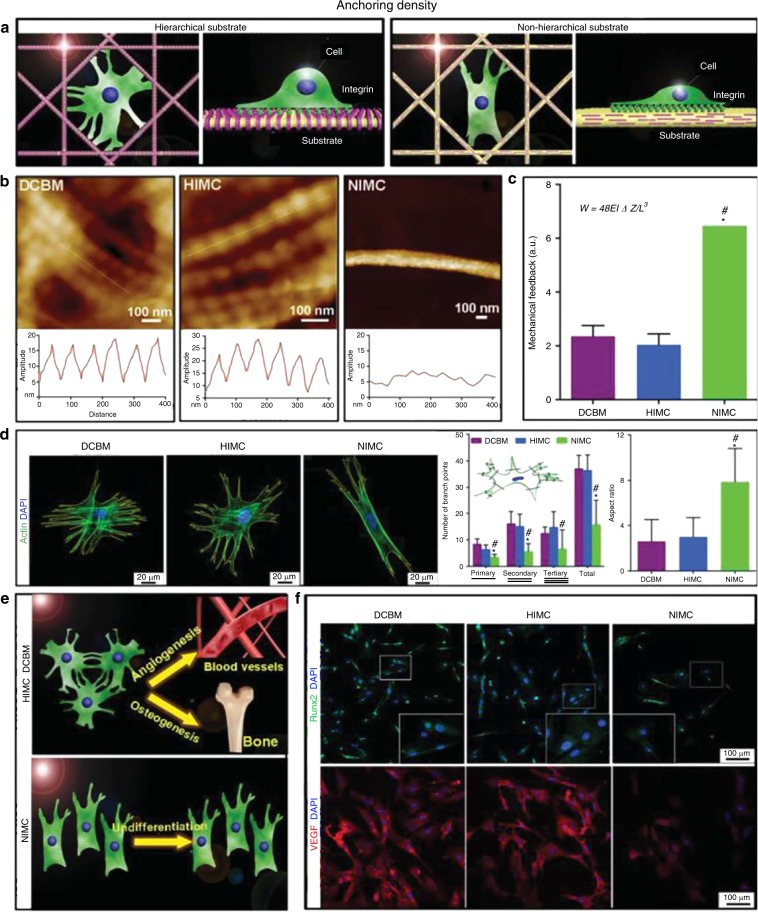
Hierarchical nanotopography regulated cell behaviors. **a** Schematic graphs demonstrating the interactions between cells and different substrate nanotopography; **b** Atomic force microscopy images showing nanotopography of decelluarized bone matrix (DCBM), HIMC and nonhierarchical intrafibrillarly mineralized collagen (NIMC); **c** Mechanical responses of different scaffolds with various nanointerfaces; **d** Substrate nanotopography of biomaterials influencing cell morphology, branch points, and aspect ratio; **e** Models of cell differential performance based on different nanointerfaces; **f** Immunofluorescent images of Runx2 and VEGF staining. (Reproduced from ref. ^27^ Copyright 2019, WILEY-VCN Verlag GmbH & Co. KGaA, Weinheim.)

A complex interplay exists between cells and nanostructures of the ECM; therefore the cell behaviors could be regulated by the nanotopology of the interface.^92,93^ As in the living organisms, cells grow in a variety of nanoscale morphological features, constructed by the folding and assembly of proteins, to achieve their biofunctions. There are some previous studies revealing how cells respond to environmental cues. For example, the ultrahigh adhesiveness of cells to certain nanostructured surfaces has been attributed to periodic arrays of hierarchical nanostructures. This nanoscale structure increases the surface area and surface wettability of the scaffold, offering favorable conditions for cell adhesion.^6^ Dalby et al. led a pioneering work to reveal the importance of nanotopographies in the population and osteospecific differentiation of MSCs. In this study, the electron beam lithography was used to fabricate different nanotopographies composed of 120-nm-diameter nanopits, including square array (SQ), displaced square array with dots deviating their position in a true square for 50 nm (DSQ 50), DSQ 20 (deviation 20 nm from true center), and random placements.^94^ After 21 days’ incubation, MSCs on the planar control SQ group showed fibroblastic appearance with no immunocytochemistry expression of osteopontin (OPN) and osteocalcin (OCN). In comparison, cells on DSQ20 possessed osteoblastic morphology with positive expression of OPN and negative expression of OCN. In the DSQ50 group, MSCs aggregated to form discrete regions and mineralized nodules at early stage, and positively expressed both OPN and OCN. Moreover, MSCs cultured on random placements showed osteoblastic morphology, but with no OPN/OCN positive expression. These results implied that the surface nanotopology induced significant differences in cellular responses. Based on this principle, we can infer that a “clever” design for materials in bone TE should be based on the simulation of natural bone’s nanotopography, that is the hierarchical nanostructure formed by staggered co-assembly of Col and nano-HA.^30^ Liu et al. used self-assembly and thermodynamic control methodologies to realize HIMC with a perfectly staggered nanostructure and nanoscale surface chemistry similar to that of natural mineralized collagen.^27,65,66^ This biomimetic nanointerface was shown to influence stem cell fate (Fig. 3)^59^ Specifically, the cells seeded on the HIMC presented a highly branched “osteocyte-like” shape, with long filopodia and a thick stress-fiber formation. Meanwhile, the expression levels of runt-related transcription factor 2 (Runx2) and VEGF in the HIMC group were much higher.^65^ Mechanically, the nanometer-scale topography in scaffolds plays a critical role in modulating cell growth and attachment, proliferation and differentiation, because the undulating changes at the nanoscale level affect on the covalent anchoring density of stem cells, thereby influencing stem cell adhesion. The relationship between anchoring density and strength of adhesion has been well explored.^27,66^ Cells react to mineralized collagen via the mechanical feedback provided by scaffolds. Hou et al. ameliorated their biomaterials by integrating protein-based nanofibrous microparticles into the injectable hydrogel to form a novel type of hydrogel.^95^ The hierarchically structured hydrogel displayed an ECM-mimicking nanofibrous architecture due to the participation of the nanofibrous microparticles. Compared with smooth surfaces, the novel hydrogel had better cell adhesion because of the higher surface area and adsorption capacity for ECM proteins such as fibronectin and vitronectin. The roughness and multiscale structural complexity of the scaffold nanostructure were thus very important for the regulation of cell activity. When it comes to cell adhesion, it is clear that integrins, as critical communication channels, involve in the responses of cells to a nanotopographical surface.^96^ Generally, biochemical regulation of cell behaviors can be achieved by controlling adhesion size and changing adhesion-related signaling, such as ERK 1/2 and c-Jun N-terminal kinase when a nanotopographical system is used.^96^ The mechanotransduction from the physical properties of biomaterial scaffolds has substantial implications for stem cell biology and bone TE. Thus, harnessing nanoscale and nanotopographic features to control MSC activity shows promising potentials for the application of bone regenerative medicine.^97^

**Fig. 4:**
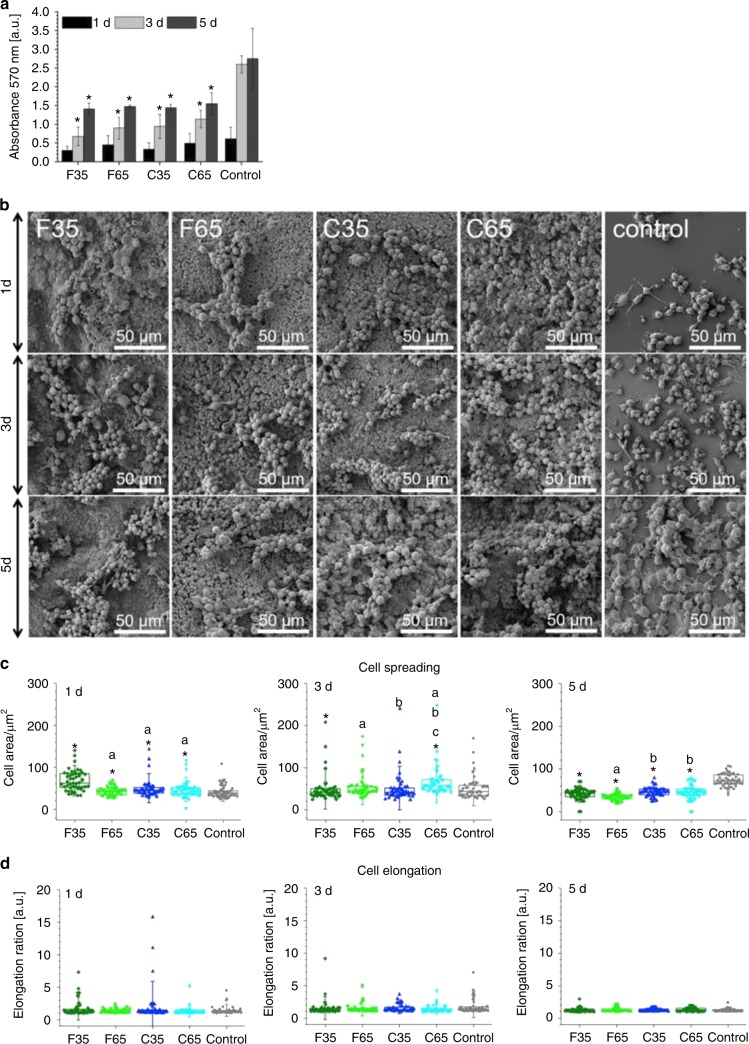
Nanotopography regulte the osteogenic processes by inducing the macrophages bebavior. **a** MTT assay of cells on CDHA at 1, 3 and 5 days; **b** SEM images of cells on CDHA at 1, 3 and 5 days; **c** Semiquantification of cell area at 1, 3 and 5 days; **d** Semiquantification of cell elongation at 1, 3 and 5 days. (Adapted with license from the ref. ^100^ Copyright 2018, Elsevier Ltd.)

Apart from regulating cell fate, the surface nanotopography of biomaterial scaffolds could orchestrate osteogenesis by modulating the local immune microenvironment. Macrophages, as the main immune cell mediating biomaterial-related response, could be regulated by nanotopographies, such as cell shape, proliferation, adhesion and phenotype.^98,99^ Joanna et al. have reported that nanostructured needle-like calcium deficient HA could facilitate osteogenesis of bone forming cells, as the surface topography of calcium deficient HA plays an important role in inducing the release of pro-inflammatory cytokines by macrophages, which in turn regulate the osteogenic processes (Fig. 4).^100^ In addition, Jin et al. also have demonstrated that the immune response of macrophages could be stimulated through some physicochemical properties of nanostructured biomaterial scaffolds.^65^ They have showed the biomimetic hierarchical nanointerface can facilitate M2 macrophage polarization and interleukin-4 secretion to promote stem cell osteogenesis and endogenous bone regeneration. Similarly, Chen et al. have shown that nanoengineered surfaces with plasma-polymerized acrylic acid and 68 nm height nanotopography could provide prime immune microenvironment for enhancing osteogenesis by inhibiting inflammation, modulating M2 macrophage polarization, regulating osteoclastic activities and expression levels of angiogenic, fibrogenic and osteogenic differentiation markers in macrophages.^101^ Besides the interactions between nanostructures and macrophages, monocyte immunomodulation also plays an important role in angiogenesis and osteogenesis in bone TE.^102,103^ Sun et al. have reported that silicified collagen nanofibers could promote monocyte recruitment and differentiation, and cytokine release to further home MSCs and endothelial progenitor cells, and therefore enhance local vascularization and bone regeneration.^102^ Taken together, nanotopography could directly modulate osteoblastic lineage cell activities to enhance osteogenic differentiation, and produce a favorable osteoimmune microenvironment in bone regeneration. It is therefore regarded as a powerful strategy for fabricating advanced bone substitute scaffolds in TE.

### From the microscale perspective

**Fig. 5:**
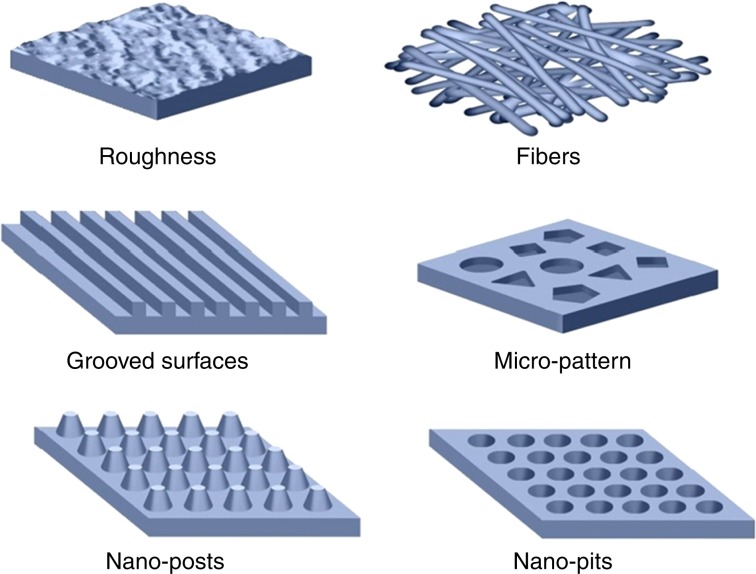
Schematic micro/nanoscale surface patterns. (Adapted with permission from the ref. ^30^ Copyright 2019, Elsevier Ltd.)

The micropattern surface, the alignment of ECM and bone cells, and the interconnected 3D pore structure are important microstructural features (Fig. 5).^30,104^ The micropattern architecture directed multicellular organization and fibrillar collagen deposition, and also affected the alignment and shape of single cells. Additionally, aligned cues were sufficient to direct cell shape, alignment, adhesion, and fibrillar collagen matrix deposition.^105^ Cells in an ordered alignment are more likely to differentiate into an osteogenic phenotype.^30^ Gilchrist et al. compared three types of scaffolds with different architectures: unpatterned, gridded, and aligned pattern.^105^ They found that single cells on aligned patterns have more higher alignment ratio than those on gridded and unpatterned substrates. The actin cytoskeleton on the aligned pattern arranged along the same direction as the pattern, and promoted the deposition of fibrillar collagen. Maleki et al. developed a biomaterial with a honeycomb-shaped micromorphology and microstructural alignment at varied length scales. They used a unidirectional freeze-casting approach with ice as the structural directing agent, and adjusted reaction parameters during fabrication process to control the micromorphology of the resulting aerogel.^106^ The microstructural pattern provided a platform for the attachment of osteoblast cells for growth, proliferation, osteogenic differentiation, mineralization, and eventual bone formation. The alignment of the materials comprised the morphological pattern of the scaffold, which was also closely related to pore formation. In this study, the aerogel was essential for the formation of the hierarchically organized porous structure. In reality, multiscale cues interact to influence cell behaviors and regulate the alignment of new tissue in a complex manner. Mechanistically, these biophysical cues from extracellular signals lead to changes of gene-, protein- or whole cell levels in response to membrane tension or fluidity caused by fluid shear stress or changes in the cell shape.^104^

Pore structure is another important aspect of bone scaffolds.^7^ The advantages of pores have been described in the numerous studies; they allow for osteoblasts migration and proliferation, the transport of nutrients and waste,^7,107,108^ and vascularization.^109^ Hence, well-interconnected pore structures could facilitate cell infiltration and the transportion of nutrient and nutritions and waste.^110^ Usually, scaffolds must be post-processed after their initial fabrication to develop a porous structure. Lyophilization,^1^ sintering,^28^ gas foaming,^111^ and phase separation^109^ are commonly used techniques for pore formation. Pore parameters such as size, porosity,^7^ and interconnectivity^109^ crucially influence cell behaviors. Generally, large pores contribute to osteogenesis by generating mineralized bone tissue because of allowing ingrowth of blood vessels and high oxygenation; small pores mainly provide more adsorption sites for bioactive molecules, and improve the nutrient and metabolic waste transport.^112,113^ The pore size should be in the range of 50 to 150 μm.^112^ Petersen et al. prepared a macroporous scaffold with uniform pore size of 89 ± 15 μm that realized higher cell migration depth.^114^ Kim et al. used 3D printing with self-setting reactions and salt leaching to engineer hierarchical scaffolds with different porosities and pore architectures. They studied the effect of pore structure on bone regeneration in vivo and found that micro-sized (<25 μm) pores can induce the maturation and remodeling of new bone.^115^ High porosity, an essential element to osteogenic outcomes, provides a larger surface area, thus a greater attachment opportunities for bone-inducing protein adsorption, ion exchange and apatite formation. Meanwhile, a scaffold with porous structure has a rough surface that favors the proliferation and differentiation of cells. Additionally, the interconnected pores provide cell ingrowth channels.^7^ Viswanathan et al. synthesized scaffold with closed and open pore structures, and found that pore interconnectivity regulated stem cell adhesion and differentiation.^113^ In the same way, Zhou et al. successfully fabricated a hierarchical interconnected pore structure with suitable porosity and pore size that enabled stem cells to proliferate and differentiate more actively, and to rapidly grow into the scaffold during the osteogenesis.^28^ Besides, the pore structure and size could affect cell shape and spreading of macrophages, modulate autophagy activation, subsequently suppress inflammatory and promote osteogenesis.^116^

## Chemical cues of scaffolds facilitating bone formation

The key cues to determine cell specific phenotypes are more than just providing a proper physical parameters with a native-like milieu, but also involve some molecular chemical signals.^117^ With the ongoing development of bone TE, modifications of biomaterial scaffolds with chemical groups or controlled growth factors could overwhelmingly enhance their physicochemical properties and endow them with satisfying biofunctions.^117^ Biomaterials have been optimized by incorporating additional chemical groups^118^ or bioactive factors,^119^ as well as by releasing certain growth factors,^120^ ions,^121^ and other novel active small molecules. To this end, there is increasing research on modifying the surface architecture and chemical components of 3D biomaterial scaffolds to enhance cell adhesion, growth, differentiation, and migration, and consequently bone regeneration.^122^ These bioactive scaffolds open a new approach for bone TE worthy of our ceaseless exploration.

### Chemical modifications on scaffold surface

**Fig. 6:**
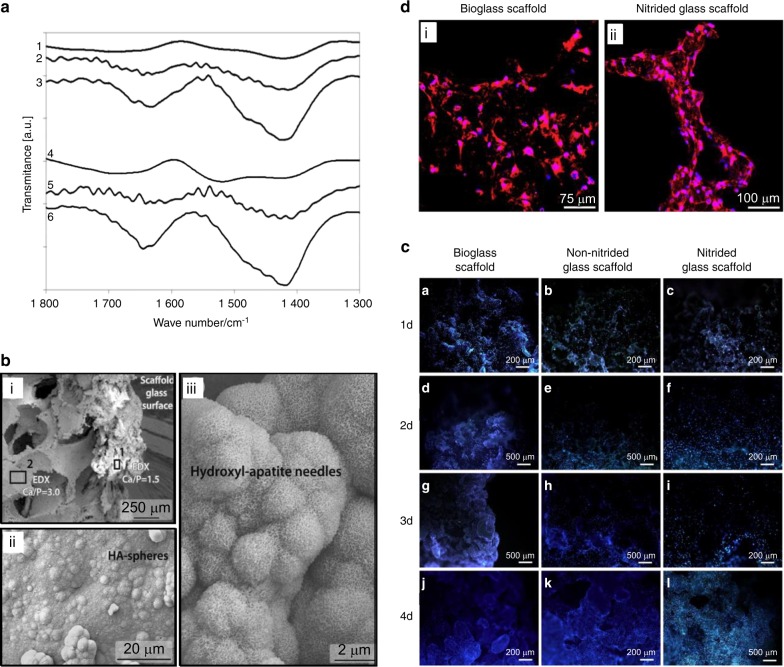
The modification of chemical groups on the surface of scaffolds influence cell adhesion and osteogenic differentiation. **a** Fouries transform infrared microspectroscopy spectra of different scaffolds: (1) non-nitrided scaffold before soaking, (2) non-nitrided scaffold after 1 day in simulated body fluid, (3) non-nitrided scaffold after 7 day in simulated body fluid, (4) nitrided scaffold before soaking, (5) nitrided scaffold after 1 day in simulated body fluid, (6) nitrided scaffold after 7 day in simulated body fluid. **b** SEM images of the nitrided glass scaffold: (i) the surface topography and the internal pores from right to left; (ii) the spherical HA structures on the surface of scaffold, (iii) the spherical HA structures formed by hydroxyl-apatite needles. **c** Hoescht nuclear staining images of osteoblastic cells on bioglasses, non-nitrided and nitrided glass scaffolds on day 1, day 2, day 3 and day 7. **d** Alkaline phosphatase staining of osteoblastic cells on bioglass scaffolds and nitrided glass scaffolds. (Reused from ref. ^123^ with permission. Copyright 2015, Acta Materialia Inc. Published by Elsevier Ltd.)

Changing chemical composition of a scaffold endows it with different biofunctions. Some researchers have explored the possibility of modifying chemical groups on the surface of a scaffold as a way to improve cell adhesion and osteogenic differentiation (Fig. 6).^123^ The enhanced stem cell adhesion might be attributed to the increased protein adsorption via the –NH_2_ and –COOH functionalized scaffolds.^118^ The researchers deposited –NH_2_ and –COOH groups on scaffolds by using allylamine and acrylic acid. They intriguingly found that the –NH_2_ modification supported osteogenesis due to the formation of hydrogen bonds between its positive charge and fibrinogen, while the –COOH group promoted chondrogenesis. Thus, compared with the –COOH group, the –NH2 group is more conducive for skeletal TE. Similarly, Yu et al. demonstrated that the −NH_2_ modified surface exhibited improved biocompatibility and osteoconductivity/osteoinductivity with increased cell adhesion and proliferation capabilities.^124^ In addition, Zamani et al. described 3D-printed PCL scaffolds that were surface-modified by alkaline treatment with 1 mol·L^−1^ and 3 mol·L^−1^ sodium hydroxide (NaOH) for 24 h.^125^ Their investigation showed that the NaOH-treated scaffolds had a honeycomb-like surface pattern, and that the increased number of hydroxyl and carboxyl groups on the surface increased hydrophilicity via scission of PCL ester bonds by NaOH. Furthermore, the scaffold post-treated by NaOH displayed increased calcium deposition. It is unequivocal that the surface topography of scaffolds affects cell behavior, ant that the surface of scaffolds can be modified to encourage osteoblast attachment and proliferation. Small changes in the surface chemistry of a scaffold prepared by Neffe et al. provided an improved microenvironment for endogenous cell recruitment, osteogenic differentiation and bone regeneration. Specifically, they fabricated a multifunctional scaffold with combination of porous interconnected architecture and chemical functionalization though controlling the ratios of diisocyanate to amino groups of gelatin. In this study, the gelatin contained in the scaffold was connected with a derivative of the amino acid L-lysine by urea junction units, without the involvement of any growth factors.^126^ This one-step synthesized multifunctional hydrogel scaffold, with a small chemical modification, possesses enhanced micromechanical properties and a proper degradation behavior, and subsequently leads to satisfying regenerative outcomes.

### Controlled release of active chemical components for bone TE

The biofunctions of bone substitute scaffolds depend on structural elements or the loaded single or dual chemical components. The synergistic effect between physical and biochemical stimuli provides additional advantages for bone regeneration.^127^ It has been shown that certain nano/microparticles could be released from biomaterial scaffolds when the scaffolds degrade. These particles contain inorganic ingredients, metal ions, miRNA, and growth factors, which could stimulate cell osteogenesis and vascularization.

The incorporation of inorganic components has been shown to optimize biomaterial performances, in terms of topography, mechanical properties, surface area and cell behaviors. For example, biosilica incorporation could increase the porosity of scaffold, initiate mineralization, and promote cell attachment and proliferation.^128,129^ The application of graphene oxide activates osteogenesis and enhances biocompatibility. The function of graphene oxide in composite scaffolds for bone regeneration is closely related with its concentration and interaction period.^130^ Xia et al. incorporated superparamagnetic iron oxide nanoparticles (γIONP) and iron oxide nanoparticles into CaP cements to fabricate magnetic scaffolds (γIONP-CPC) and nonmagnetic scaffolds, respectively.^131^ They found that the an IONP-incorporated CaP cement scaffold enhanced cell performances via an exterior static magnetic field. This novel magnetic construct is highly promising for bone regeneration, especially γIONP-CPC, because the internalized magnetic γIONPs inside the cell membrane reoriented and distorted, resulting in an alteration of the cell cycles and differentiation. Additionally, some metal ions such as calcium (Ca), magnesium (Mg),^132,133^ strontium (Sr),^121^ and copper (Cu)^134^, which mediate chemobiological homeostasis of human, are widely applied in chemical modifications on bone substitute scaffolds to stimulate the osteogenesis and angiogenesis. Minardi et al. added Mg to the HA/Col I composite and showed that cells seeded in vivo in the scaffold retained high viability and reproducibility for mature cortical bone formation.^132^ Interestingly, there are reports showing that the release of ions has only a slight but not significant effect on cell adhesion and differentiation, while the underlying material substrates are the key regulators.^135^ Therefore, Mg-doped scaffolds are considered to promote adhesion and osteogenic differentiation of MSCs by inducing the expression of integrin α5β1 receptor.^133,135^ Similarly, Ryan et al. developed a Cu-doped bioactive glass scaffold to stimulate bone regeneration.^134^ In this study, they demonstrated that Cu induced osteogenic differentiation of MSCs through promoting collagen maturation by lysyl oxidase crosslinking. Autefage et al. designed a porous scaffold to achieve controlled release of Sr, which induced tissue infiltration and encouraged bone formation.^121^

Recently, osteoimmunomodulation is getting more and more attention in bone TE, as a favorable osteoimmune microenvironment plays a vital role in successful biomaterials-mediated bone regeneration. The controlled release of certain metal ions from scaffolds, such as Zinc, Mg and Sr, could orchestrate osteogenesis by modulating the local immune microenvironment. Zinc could modulate nonactivated macrophage polarization and stimulate the release of anti-inflammatory and osteogenic cytokines.^136^ Chen et al. reported that coating Mg with -TCP could decrease degradation rate of the composite scaffold, regulate M2 macrophage polarization, promote osteogenic differentiation of MSCs and inhibit osteoclastogenesis simultaneousl.^137^ Ca and Sr are shown to induce osteogenesis and inhibit inflammation.^138^ Zhang et al. showed that incorporation of Sr into bioactive glasses synergistically enhance osteogenesis by modulating macrophage polarization.^139^ Yu et al. also demonstrated that the Sr-doped amorphous CaP porous scaffold improved new bone formation.^140^ Furthermore, synergistic biofunctions of osteogenesis and immune response are achieved by the coating of Ca and Sr with a Ca/Sr ratio of 2:1.^141^ Besides, coating of macrophage-affinitive glucomannan enhances the bone regenerative performance of 3D hydrogel scaffolds.^142^

**Fig. 7:**
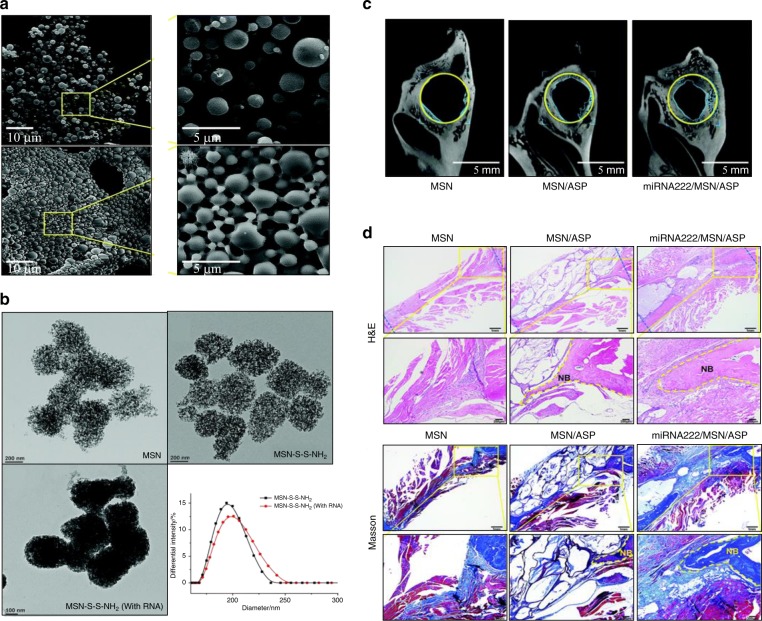
MiR-222 in the scaffold enhance tissue regeneration. **a** SEM images of microspheres in the solution and hydrogel state. **b** TEM images of porous and spherical morphology of mesoporous silica nanoparticles (MSNs) and miR222/MSNs. **c** Micro-CT images of new bone formation after implantation of MSN, MSN/ASP, and miR222/MSN/ASP microsphere hydrogels in rat mandible defects for 10 w. **d** H&E and Masson’s trichrome staining of new bone formation in different groups. (Adapted with permission from the ref. ^144^ Copyright 2019, The Royal Society of Chemistry.)

MiRNAs are being developed to enhance tissue regeneration, because they can downregulate or upregulate the expression of their target genes.^143^ Zhang et al. showed that miR-26a increased osteoblast activities by functionally targeting Gsk-3β in bone repair.^4^ Lei et al. fabricated an injectable colloidal hydrogel with mesoporous silica nanoparticles loaded miR-222 and aspirin (ASP). They found that the miR-222 in the scaffold promote neurogenesis and bone regeneration via inducing neural differentiation in bone marrow MSCs (Fig. 7).^144^ Theoretically, with the exception of ions and miRNA, bioactive factors that are involved in skeletal development and remodeling, such as transforming growth factors, BMPs, fibroblast growth factors, and Runx2 can also be used to improve scaffold biofunctions.^145^ BMP-2 is the most commonly studied factor; it can be introduced to different scaffolds by various techniques, and can be embedded in cell-derived ECM to provide an osteogenic microenvironment with slow release of BMP-2.^146^ Additionally, Li et al. encapsulated BMP-2 into bovine serum albumin to maintain the bioactivity of BMP-2, and thus achieved the osteogenic differentiation and osteogenesis.^89^ Surface coating is another route to trap and subsequently release BMPs to trigger rapid volumetric bone regeneration.^120^ Multiple growth factors can also be exposed on the scaffold surface to promote new bone formation.^147^ For example, FGF2 is favor of cell migration, whereas VEGF promotes vascularization at the defect site. Thus, incorporation of FGF2 and VEGF into biomaterial scaffolds not only promotes recruitment of endothelial stem cells and MSCs, but also facilitates the new blood vessel formation during bone regeneration.^148^ The combination of multiple growth factors provides a promising strategy for bone regeneration, However, the dose of each growth factor and the duration time of growth factor release are unclear and need further exploring.

## Conclusions and perspectives

Current approaches for bone TE involve three elements: scaffolding biomaterials, cells with osteogenic ability, and growth factors. The nano/micro architecture of biomimetic 3D biomaterial scaffolds provides a suitable microenvironment for skeletal regeneration. Nanotopography could directly modulate osteoblastic lineage cell activities to enhance osteogenic differentiation, and produce a favorable osteoimmune microenvironment. The microstructural pattern could provide a platform for the attachment of osteoblastic lineage cell for growth, proliferation, osteogenic differentiation, mineralization, and eventual bone formation. It is clear that chemical signals can facilitate bone formation by regulating osteogenic gene expression. There is a growing emphasis on multifunctional scaffolds loaded with various nanoparticles or molecules for use as tools to concurrently stimulate the proliferation and differentiation of osteoblastic lineage cells. However, despite the extensive research reported to date, the mechanisms of nano/micro-assisted strategies for bone TE are poorly understood and need further study.
